# Laparoscopic distal gastrectomy for advanced gastric cancer with situs inversus totalis: a case report

**DOI:** 10.1186/s40792-022-01532-4

**Published:** 2022-09-27

**Authors:** Shunsuke Fujita, Tsuyoshi Etoh, Yohei Kono, Hajime Fujishima, Kosuke Suzuki, Shigeo Ninomiya, Yoshitake Ueda, Hidefumi Shiroshita, Norio Shiraishi, Masafumi Inomata

**Affiliations:** 1 Department of Gastroenterological and Pediatric Surgery, Oita University Faculty of Medicine, 1-1 Idaigaoka, Hasama-machi, Oita, 879-5593 Japan; 2 Department of Advanced Medical Research and Development for Cancer and Hair, Oita University Faculty of Medicine, Oita, Japan; 3 Department of Comprehensive Surgery for Community Medicine, Oita University Faculty of Medicine, Oita, Japan

**Keywords:** Situs inversus totalis, Advanced gastric cancer, Laparoscopic gastrectomy

## Abstract

**Background:**

Situs inversus totalis (SIT) is a relatively rare condition, in which the thoracic and abdominal organs are reversed or mirrored from their normal positions. Here, we reported a case of a patient with SIT and advanced gastric cancer with lymph node metastasis who underwent laparoscopic distal gastrectomy (LDG).

**Case presentation:**

A 67-year-old man with SIT was clinically diagnosed with T3N2M0 advanced gastric cancer located in the middle gastric body. Three-dimensional reconstruction of computed tomography angiogram revealed that the common hepatic artery originated from the superior mesenteric artery. The patient underwent LDG with D2 lymph node dissection and Roux-en-Y reconstruction. The postoperative course was uneventful.

**Conclusion:**

This case report showed that LDG could be safely performed on a patient even under complex conditions, such as advanced gastric cancer with lymph node metastasis with SIT and vascular anomalies.

## Background

Situs inversus totalis (SIT) is a relatively rare autosomal recessive congenital anomaly found in one per 8000 to 25,000 persons [1] and is characterized by the position of the cardiopulmonary and abdominal organs being inverted. Although a recent randomized controlled trial (RCT) revealed that laparoscopic gastrectomy for gastric cancer had become a standard procedure especially for clinical stage I cancer [2], this procedure is technically demanding owing to the anatomical anomalies in patients with SIT. There are several reports on laparoscopic gastrectomy for SIT; however, only a few reports have presented laparoscopic gastrectomy for patients with advanced gastric cancer with SIT [3–6], which is considered a complex procedure because of the additional requirement for D2 lymph node dissection. Previous reports regarding the management of advanced disease pertained to patients with SIT who did not have lymph node metastases on clinical assessment. Here, we report a case of laparoscopic distal gastrectomy (LDG) with therapeutic D2 lymph node dissection for clinical metastatic lymph nodes and Roux-en-Y reconstruction for advanced gastric cancer with SIT and review the previous literature on this subject.

## Case presentation

**Fig. 1:**
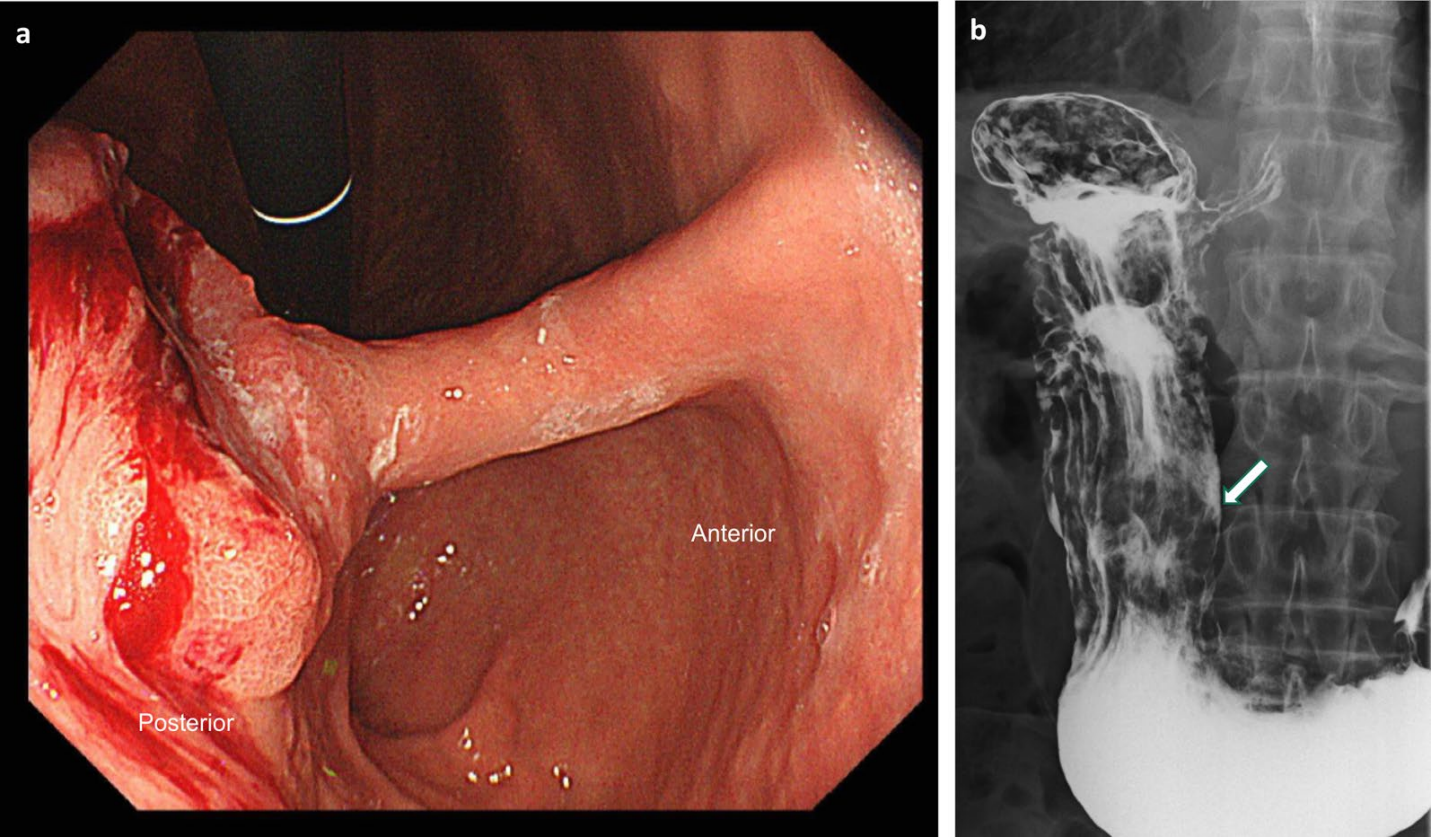
Preoperative imaging. **a** Gastrointestinal endoscopy findings. A type 2 tumor was found on the posterior wall of the middle gastric body. **b** Double-contrast barium imaging. An elevated tumor with ulcerative lesions of 4 cm diameter was detected in the posterior wall of the middle gastric body (arrow)

**Fig. 2:**
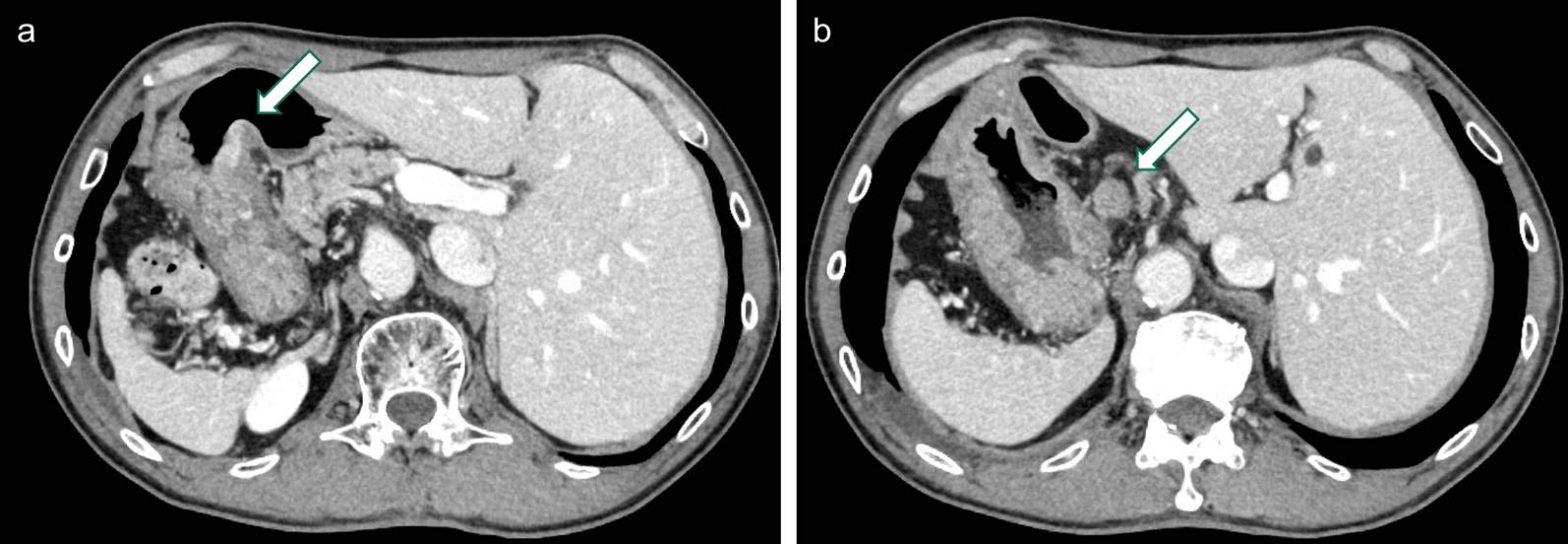
Computed tomography (CT) findings. CT showed complete transposition of the abdominal organ, irregular thickening of the gastric wall (**a**, arrow), and metastasis to lymph node #7 (**b**, arrow)

**Fig. 3:**
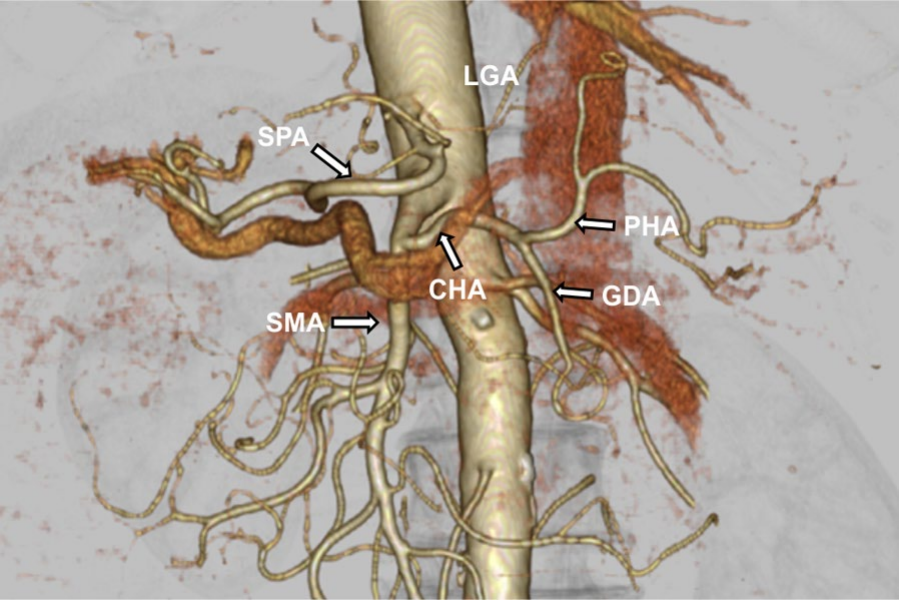
Three-dimensional reconstruction image of CT angiography preoperative findings. The common hepatic artery (CHA) originated from the SMA. *CHA* common hepatic artery, *SPA* splenic artery, *SMA* superior mesenteric artery, *LGA* left gastric artery, *PHA* proper hepatic artery, *GDA* gastroduodenal artery

A 67-year-old man with SIT was diagnosed with advanced gastric cancer using Gastrointestinal endoscopy, performed at an outside hospital during investigation for epigastric discomfort. The patient was subsequently referred to our hospital for further evaluation. He was diagnosed with SIT at the age of 17 years. Gastrointestinal endoscopy showed an elevated lesion with an ulcer (type 2) on the posterior lower body of the stomach (Fig. 1a), and histological examination revealed moderately differentiated tubular adenocarcinoma. Double-contrast imaging revealed a lesion in the deformed posterior wall of the middle gastric body (Fig. 1b). Additionally, computed tomography (CT) revealed inverted abdominal organs and irregular thickening of the gastric wall with swollen lymph nodes and no distant metastasis (Fig. 2a, b). A three-dimensional (3D) reconstruction image of CT angiography showed complete transposition of vessels and branching of the common hepatic artery (CHA) directly from the superior mesenteric artery (SMA) (Fig. 3). The tumor marker levels were within the normal ranges. The patient was preoperatively diagnosed with a clinical T3N2M0 Stage IIIA according to the third edition of Japanese Classification of Gastric Carcinoma [7]. Consequently, LDG with D2 lymph node dissection and Roux-en-Y reconstruction were planned.

**Fig. 4:**
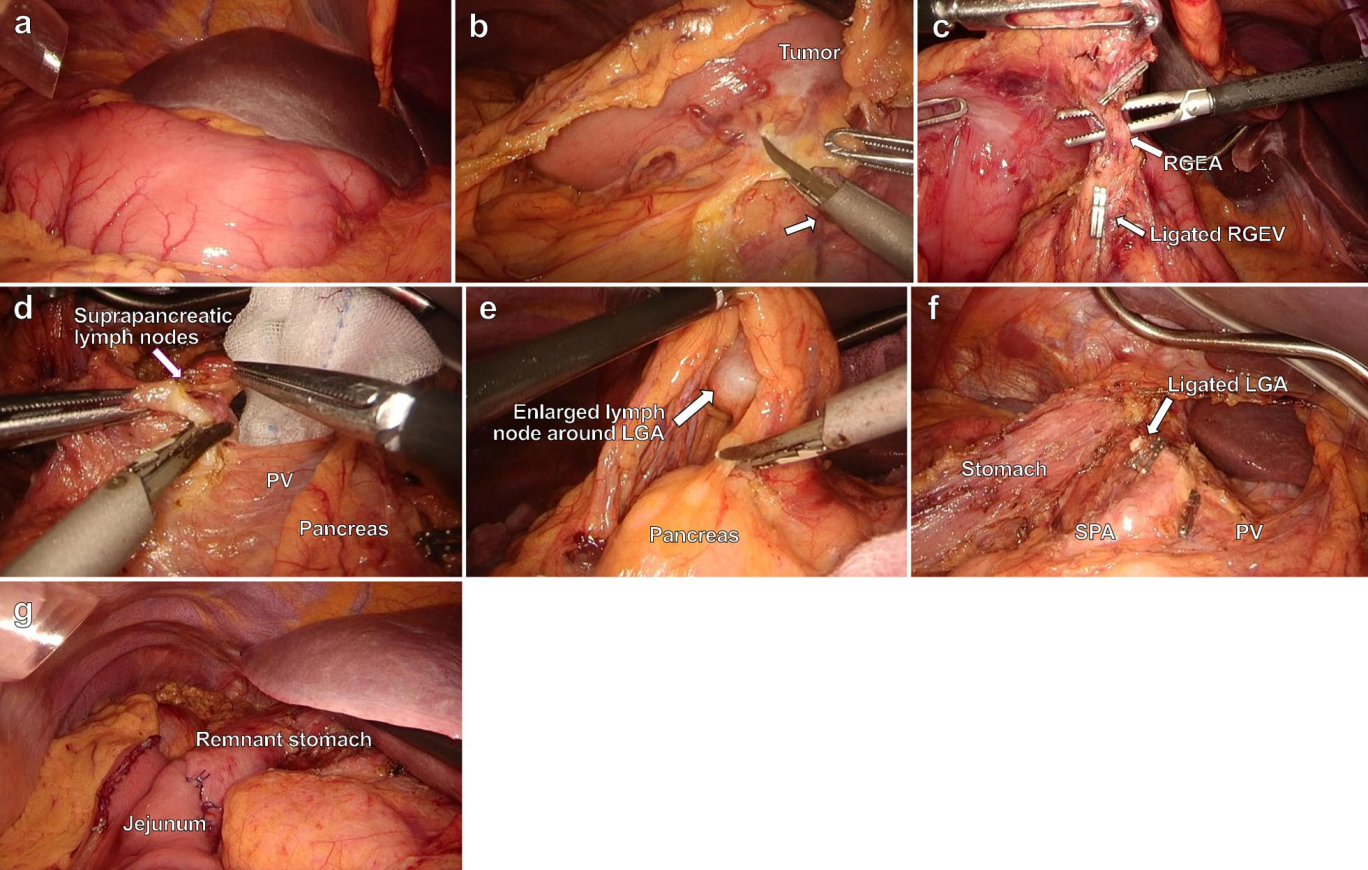
Intraoperative findings during laparoscopic distal gastrectomy. **a** Laparoscopic view showing inversion of the abdominal organs. **b** The energy device was used by the non-dominant hand of this operator (arrow). **c** In the infrapyloric area, RGEV and RGEA were ligated, respectively. **d** On the left side of the suprapancreatic area, the anatomical landmarks of lymph node dissection were the upper border of the pancreas, PV. **e** Enlarged lymph node of 3 cm in diameter was found around the LGA. **f** Completion of lymph node dissection in the suprapancreatic area and lesser curvature side of the stomach. **g** Roux-en-Y reconstruction. *RGEV* right gastroepiploic vein, *RGEA* right gastroepiploic artery, *LGA* left gastric artery, *PV* portal vein, *SPA* splenic artery

Surgeons with the Endoscopic Surgical Skill Qualification System (ESSQS) [8] accreditation participated in this operation. During the operation, the surgeon stood on the left side of the patient, opposite the usual side for surgery at our hospital. Four trocars were inserted in the left and right subcostal and lateral abdominal regions. The laparoscopic view showed an inversus of the intra-abdominal organs, including the stomach and the spleen (Fig. 4a). After the position of the spleen was confirmed, ligation of the left gastroepiploic vessels with partial omentectomy was performed. During the operation, the usage of the dominant hand was often the opposite of the usual manner. For example, the non-dominant hand of this operator handled the energy device shown in Fig. 4b. While dissecting the region around the right gastroepiploic vessels, the surgeon moved to the right side. However, we found that sometimes it was technically easier to perform this dissection from the opposite side, and the surgeon occasionally alternated between the right and left sides. In lymph node dissection of the infrapyloric area, the pancreas head, duodenum, and transverse colon mesenterium are considered anatomical landmarks. Therefore, each landmark was confirmed during the procedure, and finally the right gastroepiploic vein and artery were identified and ligated (Fig. 4c).

Next, the surgeon moved to the left side, and the lateral segment of the liver was retracted instrumentally. The lesser omentum was resected, and the right gastric artery from the proper hepatic artery (PHA) was identified and ligated. As this patient had the CHA originating from the SMA, the anatomical landmarks of lymph node dissection in the suprapancreatic area were considered the upper border of the pancreas, portal vein, and left gastric artery (LGA) from the celiac axis (Fig. 4d). In addition, an enlarged lymph node of 3 cm diameter was found along with the coronary vein and LGA and therapeutic lymph node dissection was performed without touching the enlarged lymph node (Fig. 4e). Then, lymph node dissection was performed along the dorsal plane of the proximal splenic artery (SPA) and was performed for another enlarged lymph nodes along the lesser curvature up to the esophagogastric junction (Fig. 4f). Finally, a Roux-en-Y antecolic gastrojejunostomy was performed under minilaparotomy (Fig. 4g). Notably, the operation time was 446 min, and the blood loss was 3 mL.

In the resected specimen, it was observed that the tumor was 30 × 21 mm in diameter, and pathological examination revealed a moderately differentiated tubular adenocarcinoma with subserosal invasion and three metastatic lymph nodes. The final pathological stage was pT3N2M0 stage IIIA according to the third edition of Japanese Classification of Gastric Carcinoma [7]. The postoperative course was uneventful, and the patient was discharged at 14 days after surgery. Notably, the patient was administered adjuvant chemotherapy with oral anticancer agents. There is no evidence of recurrence at 6 months postoperatively.

## Discussion

The occurrence of advanced gastric cancer in patients with SIT is rare. As the intra-abdominal anatomy is complex for surgery in SIT cases, laparoscopic gastrectomy is a technically demanding procedure, especially for advanced gastric cancer cases. Therefore, using Medline and PubMed databases for case reports, we searched for literature published in the English language from 2000 to 2022 with the following keywords: “advanced gastric cancer”, “laparoscopic gastrectomy”, and “situs inversus totalis”. The characteristics of patients with SIT and advanced gastric cancer who underwent laparoscopic gastrectomy were found in four previous reports. These case reports are listed in Table 1 [3–6]. Three of the listed cases had vessel abnormalities. All of the previous reports showed clinical absence of lymph node metastases and D2 lymph node dissection. Our case had a longer operation time compared with previous reports; on the other hand, the patient had minimum blood loss, less than 10 mL. The postoperative course in each case was uneventful.

**Table 1:** Characteristics of patients of inversus totalis with advanced gastric cancer who underwent laparoscopic gastrectomy

Authors	Year	Age/sex	cT	cN	pStage	Vessel anomalies	Position of the surgeons	Type of gastrectomy	Lymph node dissection	Reconstruction	Operation time (min)	Blood loss (mL)	Complications
Min et al. [3]	2013	52/M	T2	N0	IB	CHA from SMA	Same as ordinal	Distal	D2	Billroth I	220	100	None
Ye et al. [4]	2015	60/M	T3	N0	IIB	None	Opposite	Distal	D2	Billroth II	230	50	None
Shibata et al. [5]	2016	79/M	T3	N0	IIB	None	Same as ordinal	Total	D2	Roux-en Y	232	110	None
Namikawa et al. [6]	2021	74/M	T2	N0	IB	CHA from SMA	Opposite	Distal	D2	Roux-en Y	335	20	None
The present case	2022	67/M	T3	N2	IIIA	CHA from SMA	Opposite	Distal	D2	Roux-en Y	446	3	None

TNM staging according to the 14th edition of Japanese Classification of Gastric Carcinoma

*cT* clinical T, *cN* clinical N, *pStage* pathological stage

According to the latest Japanese gastric cancer treatment guidelines, laparoscopic gastrectomy for clinical stage I cancer has become a standard treatment in clinical trials [2, 9]. Moreover, D2 lymph node dissection is considered a standard procedure [10, 11]. Recent RCTs from Japan and Eastern Asia demonstrated the technical feasibility of laparoscopic gastrectomy with D2 lymph node dissection for advanced gastric cancer treatment [12–14]. In addition, as the efficacy of this procedure has been proven in these trials [15, 16], it could be a standard treatment.

In patients with SIT and advanced gastric cancer, there are several concerns regarding the safety of laparoscopic gastrectomy. First, anatomical variations, such as vessels and visceral organs, are known to make this procedure challenging. Therefore, an accurate understanding of surgical anatomy is necessary before surgery. Previous reports have shown that 3D CT angiography is a useful modality for confirming surgical anatomy [3–5]. In our case, 3D CT clearly demonstrated that the CHA directly originated from the SMA. Based on the preoperative information, we performed adequate D2 lymph node dissection of the metastatic lymph nodes, despite the vessel abnormality. In addition, malformation of the small intestine, which is often observed in patients with SIT, may influence the complexity of Roux-en-Y reconstruction. In this case, preoperative simulation using 3D CT helped us understand the mesentery of the small intestine loops.

Second, the possibility of varying the surgical procedures, such as trocar position, surgeon’s positioning and usage of energy device is a concern, although the oncological concept is not different even in an SIT case. The fact that our patient had clinically metastatic lymph nodes added to the technical difficulties. An accurate anatomical understanding is essential to secure a safe operative field. For example, the lower border of the pancreas, transverse mesocolon, right epiploic vessels, and duodenum are considered landmarks for lymph node dissection in the infrapyloric area. In the suprapancreatic area, the upper border of the pancreas and the outermost layer around the major vessels are the landmarks. Notably, the mirror image led to inconvenient maneuvers for the operator and assistants in this case; however, we frequently changed our position to secure the appropriate operation field and recognition of surgical landmarks. In addition, the usage of the dominant hand may often be the opposite of the usual manner. In our case, the non-dominant hand was used to hold the forceps or energy devices in some parts of the operation as planned. Despite the above measures, the operation time in this case was higher than that in the case without SIT. Frequently changing position of either operator or assistant to find anatomical landmarks and secure appropriate operation field during operation for SIT cases may need more time. Regarding reconstruction, the extracorporeal Roux-en-Y method was selected owing to tumor location and small size of the remnant stomach. Although there are possible reasons of the longer operation time, we should make efforts to reduce the operation time in the next SIT case based on sufficient review of this case.

Furthermore, the surgical team that will perform this operation seems to be an important consideration. In Japan, the endoscopic surgical skill qualification system (ESSQS) was established in 2004 to maintain laparoscopic technical skills and a standardized laparoscopic surgery process. Recent studies have shown that ESSQS-certified surgeons are more likely to deliver favorable outcomes after laparoscopic gastrectomy for gastric cancer [17, 18]. Therefore, it is preferable that ESSQS-certified surgeons participate in cases of advanced gastric cancer with SIT to perform the surgery safely. The operation was completed without any intra- or post-operative complications in our case.

Robotic gastrectomy has been introduced and is rapidly advancing in practice to overcome the range-of-motion limitation in laparoscopic gastrectomy for gastric cancer. Recent reports have demonstrated the technical advantages of robotic gastrectomy in patients with SIT [19, 20]. First, it is not necessary to change the position of surgeons during operation. Second, the surgeon can use the instrument with the non-dominant hand while having the same feeling as using the dominant hand. In contrast, the mirror image on abnormal surgical anatomy remains a concern. Notably, it is essential to select either laparoscopic or robotic surgery based on the understanding of the advantages and disadvantages of each modality.

## Conclusion

This case report showed that LDG could be safely performed on a patient, even under complex conditions, such as advanced gastric cancer with lymph node metastasis with SIT and vascular anomalies.

## Data Availability

All data generated during this study are included in this article.

## Abbreviations

SIT: Situs inversus totalis
RCT: Randomized controlled trial
LDG: Laparoscopic distal gastrectomy
CT: Computed tomography
3D: Three-dimensional
CHA: Common hepatic artery
SMA: Superior mesenteric artery
LGA: Left gastric artery
PHA: Proper hepatic artery
GDA: Gastroduodenal artery
SPA: Splenic artery
